# Resolving the intrinsic short-range ordering of K^+^ ions on cleaved muscovite mica

**DOI:** 10.1038/s41467-023-35872-y

**Published:** 2023-01-13

**Authors:** Giada Franceschi, Pavel Kocán, Andrea Conti, Sebastian Brandstetter, Jan Balajka, Igor Sokolović, Markus Valtiner, Florian Mittendorfer, Michael Schmid, Martin Setvín, Ulrike Diebold

**Affiliations:** 1 Institute of Applied Physics, TU Wien, Wiedner Hauptstraβe 8-10/E134, 1040 Vienna, Austria; 2 Department of Surface and Plasma Science, Charles University, V Holesovickach 2, 180 00 Prague, Czech Republic

**Keywords:** Surfaces, interfaces and thin films, Atomic force microscopy

## Abstract

Muscovite mica, KAl_2_(Si_3_Al)O_10_(OH)_2_, is a common layered phyllosilicate with perfect cleavage planes. The atomically flat surfaces obtained through cleaving lend themselves to scanning probe techniques with atomic resolution and are ideal to model minerals and clays. Despite the importance of the cleaved mica surfaces, several questions remain unresolved. It is established that K^+^ ions decorate the cleaved surface, but their intrinsic ordering – unaffected by the interaction with the environment – is not known. This work presents clear images of the K^+^ distribution of cleaved mica obtained with low-temperature non-contact atomic force microscopy (AFM) under ultra-high vacuum (UHV) conditions. The data unveil the presence of short-range ordering, contrasting previous assumptions of random or fully ordered distributions. Density functional theory (DFT) calculations and Monte Carlo simulations show that the substitutional subsurface Al^3+^ ions have an important role for the surface K^+^ ion arrangement.

## Abstract (web summary)

Mica is a naturally occurring 2D mineral that has been heavily studied in many diverse areas. Here authors present atomic force microscopy images to study the mica surface in ultra-high vacuum conditions; they unveil the distribution of its surface K^+^ ions and give insights into the distribution of subsurface Al^3+^ ions.

## Introduction

**Fig. 1:**
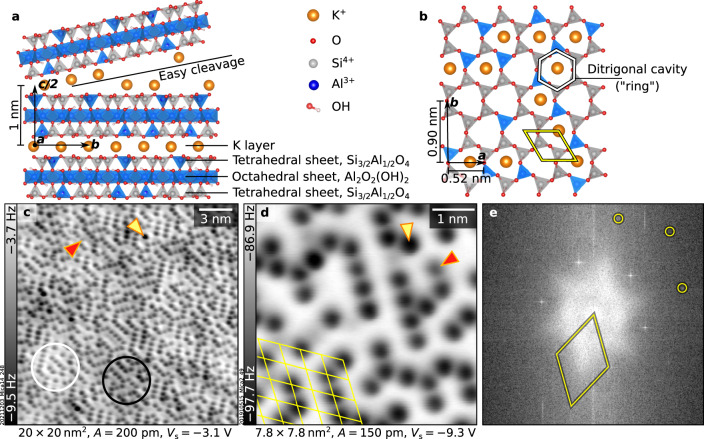
Cation ordering on as-cleaved mica. **a**, **b** Crystal structure of mica. Al ions (blue) in the tetrahedral sheets are placed in a pseudo-random arrangement akin to Fig. 4e, showing one possible arrangement fitting the experimental data. **a** Side view of bulk mica. Cleaving occurs at the K layer, leaving half the K^+^ cations on each side. **b** Top view of the surface after cleaving. Before cleaving, each ditrigonal cavity (ring, highlighted in white) is occupied by one K^+^ ion. After cleaving, 50% K^+^ ions remain on each cleaved surface. **c**, **d** Atomically resolved constant-height nc-AFM images of mica after UHV cleaving, acquired with a CO-functionalized tip and a metal tip, respectively. The images were acquired at 4.7 K with different qPlus sensors and on different samples. Yellow (red) arrows highlight species with darker (fainter) contrast than average. **e** Fourier Transform of the image shown in panel (**c**). Yellow circles mark selected diffraction spots of the underlying bulk. Unit cells of the (almost) hexagonal lattice in panels (**b**), (**d**), and (**e**) are highlighted in yellow (strictly speaking, the muscovite unit cell is rectangular because the tetrahedral rings are not perfect hexagons).

The importance and popularity of muscovite mica in surface and interface science can hardly be exaggerated. Over the last decades, the surface of this material has been the focus of hundreds of theoretical and experimental studies across diverse fields^1,2^ including environmental science, bio- and geo-chemistry, nanotribology, new-generation electronics based on 2D materials, and thin-film growth. Muscovite mica (mica, hereafter) is a common phyllosilicate with a nominal composition of KAl_2_(Si_3_Al)O_10_(OH)_2_ and a layered structure with alternating aluminosilicate and K^+^ layers (Fig. 1a, b). Each aluminosilicate layer is made of three sheets: one with AlO_6_ octahedra and OH groups (octahedral sheet), sandwiched by two sheets of 75% SiO_4_ and 25% AlO_4_ tetrahedra (tetrahedral sheet). The tetrahedra in the tetrahedral sheet form a distorted hexagonal structure, where each ditrigonal cavity (ring) hosts one K^+^ ion that compensates for the formal −1 charge introduced by the substitutional Al ions. The material easily splits apart at the K^+^ layers, which leaves half of the K^+^ ions on each created surface and maintains charge neutrality.

To the surface scientist’s delight, cleaved mica surfaces are atomically flat and virtually free of steps^3^. This, together with the broad available knowledge of its bulk properties^1^, has made mica a widely used model substrate in many contexts. Prominent examples are studies on the adsorption and dynamics of biomolecules^4–7^, motivated by the suggestion that life originated between mica sheets^4^, and studies on water^8–13^, to model the atomic-scale mechanisms underlying the water-mineral interaction that is ubiquitous on the Earth. On a more technical side, mica’s facile preparation and flatness have made it the test system of choice for emerging experimental techniques with real-space, molecular resolution in ambient or liquid conditions beyond the traditional atomic force microscope (AFM), such as the 3D AFM^14–17^.

Despite the vast popularity of mica, open questions about the system remain. Its surface K^+^ ions are central to many studies, as they can be easily exchanged in solution^10,18–23^ and offer an exciting playground to investigate ion hydration^8,24–27^ and ice nucleation^12,13,28^ on mineral surfaces. Yet, the intrinsic K^+^ distribution at the surface—unaffected by the interaction with the environment—is unknown due to the absence of UHV direct imaging. AFM images of the mica surface have been obtained in air or solution^15,24–26,29–34^, showing the cation hydration structures in these environments. However, these arrangements do not necessarily correspond to the intrinsic ones. In the ambient, the ready adsorption of water and airborne impurities^2,35^ modifies the ion–ion interaction and may promote their mobility^33^, in turn modifying the ions’ arrangements. In solution, the distribution of the hydrated ions may be affected by their increased mobility^20^, ion–water and water–water interactions^24^, and the pH. The measured arrangements have been explained in terms of water-mediated ion–ion interactions^24,33^, while the potential role of the aluminosilicate subsurface has not been considered or deemed negligible^24^. As shown in this work, however, this assumption should be revised.

Doubts exist not only on the surface K order but also on the Al order in the subsurface tetrahedral sheets. The Al distribution is hard to determine experimentally since Al and Si have similar scattering factors in X-ray diffraction. Early nuclear magnetic resonance (NMR) combined with Monte Carlo (MC) simulations^36,37^ have suggested the presence of Al short-range ordering. Such ordering could affect the distribution of the surface K^+^ ions through electrostatic interaction. Testing this hypothesis in the ambient or solution is, however, difficult for the reasons listed above.

Imaging the mica surface under ultra-high vacuum (UHV) should be well suited to assess the intrinsic ordering of the K^+^ ions and potentially relate it to the distribution of the subsurface Al ions. However, so far, individual K^+^ ions could not be resolved because UHV cleaving often introduces strong electrostatic fields that make AFM imaging challenging^38^. To the best of the authors’ knowledge, the only account of K^+^ ordering after UHV cleaving comes from low-energy electron diffraction (LEED)^39^, which has suggested a random distribution. Instead, the present results—based on non-contact (nc) AFM acquired on UHV-cleaved, clean mica—show that its surface K^+^ ions are arranged with short-range order. The distribution is analyzed with density functional theory (DFT) calculations and MC simulations, which demonstrate a close relation to the subsurface Al arrangements.

## Results and discussion

Figure 1c, d shows atomically resolved images of the mica surface after UHV cleaving. The images were acquired using the qPlus sensor^40,41^, which is stiffer (2000–3500 N/m) than standard AFM cantilevers. Hence, it is less affected by the long-range interactions with the highly charged surface of mica^42,43^ that otherwise hamper atomic contrast^38^. The images reveal an array of isolated, round, dark features arranged on a hexagonal lattice. The dark contrast represents attractive interaction between the nc-AFM tip and the sample (negative frequency shift). In the background, regions of different contrast of a few nanometers in width are visible (two of them are marked by a white and a black circle in Fig. 1c). The background contrast variation suggests an inhomogeneous long-range interaction with the AFM tip, possibly originating from trapped subsurface charges. This is also evidenced by frequency shift curves acquired as a function of tip-sample distance and sample bias (Supplementary Note 4).

The isolated dark features in Fig. 1 are assigned to the K^+^ ions left on the surface after cleaving. The features sit on a hexagonal lattice with the expected lattice constant of 0.52 nm, as determined by the Fourier transform of Fig. 1e (see Supplementary Note 5 for the analysis of the diffuse background of the Fourier transform). As expected from electrostatic considerations, the cations occupy approximately half (precisely 47.8 ± 0.1%) of the surface sites. Supplementary Note 5 discusses how the coverage was derived and why it differs from the expected 50%. It is worth noting that the exclusively attractive interaction between isolated, undercoordinated cationic adatoms on the surface and the tip is typical in nc-AFM^44,45^. The K^+^ ions cannot be deliberately nor inadvertently manipulated with the AFM tip, and their relatively large height hampers the resolution of the underlying aluminosilicate sheet.

**Fig. 2:**
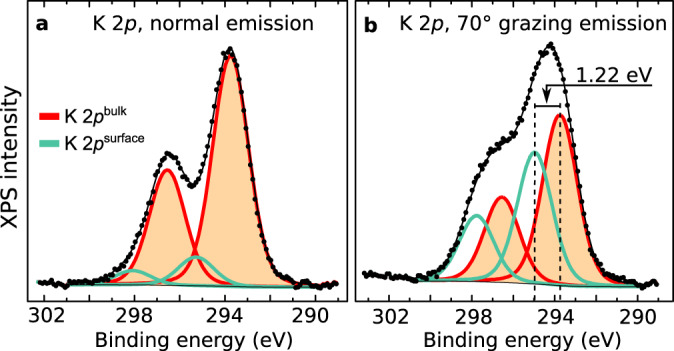
XPS spectra of K+ ions on UHV-cleaved mica. XPS data (black points) and fitted curves (solid lines) of the K 2*p* core levels (Al Kα, pass energy 20 eV). **a**, **b** Spectra taken in normal and 70° grazing emission, respectively. Two sets of K 2*p* components fit the spectra, assigned to K residing in the bulk (orange) and on the surface (green). The binding energy axes were adjusted to account for charging (see “Methods”).

An X-ray photoelectron spectroscopy (XPS) analysis further supports the assignment of the dark features to K^+^ ions. The XPS survey and the C 1*s* region in Supplementary Fig. 5 show that UHV-cleaved mica is clean except for typical substitutional trace impurities (Fe, Mg, and Na)^20,35^ listed in the supplier’s datasheet. Moreover, comparing the K 2*p* spectra acquired in normal and grazing emission (Fig. 2a, b, respectively) points to the presence of K at the surface. The marked difference in the shape of the profiles (less pronounced separation in grazing emission) is rationalized considering that XPS simultaneously probes two types of K^+^ ions: the three-fold-coordinated surface ions and the six-fold-coordinated bulk ions. Because of their different coordination, these species appear as two spin-orbit doublets with different core-level shifts. The surface component is more pronounced in grazing emission (≈43.5% of the total peak area) than in normal emission (≈11%) because of the larger surface sensitivity of the former acquisition mode. The measured separation between bulk and surface components, 1.22 eV, is reproduced by the calculated initial-state difference of 1.14 eV between the core levels originating from the surface and bulk K^+^ ions.

A small fraction of the dark species in Fig. 1c, d appear with slightly different size and contrast than the rest (arrows highlight examples). Such contrast variations are expected if atoms are located at different heights or if they are different chemical species. As discussed in Supplementary Notes 1 and 5, the species with increased contrast could correspond to K^+^ ions sitting on defective aluminosilicate rings characterized by 3 Al ions (protruding ≈0.1 Å more than K^+^ ions sitting on regular sites, according to DFT); they could also be Ca^2+^ substitutional impurities, whose higher charge compared to the K^+^ ions should cause a stronger interaction with the AFM tip. On the other hand, the fainter species are likely to be Na^+^ ions, characterized by a smaller ionic radius than K^+^ ions.

**Fig. 3:**
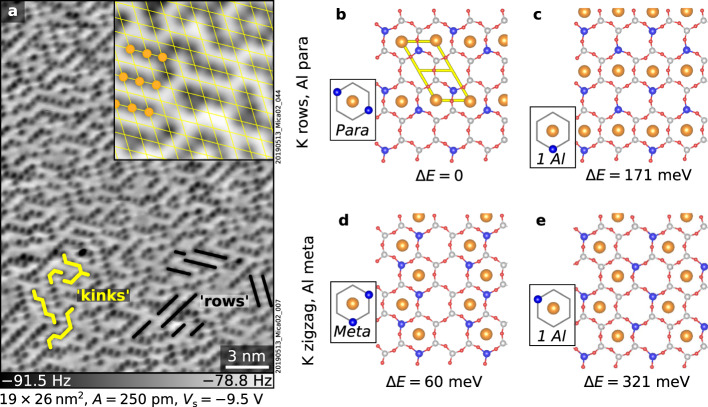
Distributions of surface K+ and subsurface Al3+ ions. **a** nc-AFM images of a UHV-cleaved mica surface. Ions arranged in alternating rows and 120° kinks, the most common arrangements, are marked in black and yellow, respectively. The 5 × 5 nm^2^ inset highlights the hexagonal lattice of mica (yellow) and local K^+^ order as alternating rows (orange). **b**–**e** DFT-calculated surface structures viewed from above (K^+^ ions: orange, Al^3+^ ions: blue; Si^4+^ ions: gray; see Supplementary Fig. 1 for the complete set of structures). They differ in the ordering of the surface K^+^ (rows or zigzag) and subsurface Al^3+^ ions (meta or para), and the number of the Al^3+^ ions in the K-occupied rings (see insets). Surface energy differences (Δ*E*) per K^+^ ion referred to the model in panel (**b**) are shown at the bottom.

The high resolution of the nc-AFM images in Figs. 1 and 3a affords detailed insights into the surface K^+^ ion ordering. The ions arrange with short-range order, forming alternating rows along the three low-index directions with an average length of 3.5 ± 0.4 nearest neighbors (NN), exemplified by the black lines in Fig. 3a. These rows are often interrupted or joined by 120° kinks of three NN, some of which are marked in yellow in Fig. 3a. The short-range order is quantified by the autocorrelation analysis in Supplementary Note 5.

What is the origin of the measured short-range order of the surface K^+^ ions? It is natural to expect that the repulsion between the K^+^ ions plays an important role. Another expected contribution is the electrostatic interaction between surface K^+^ and subsurface Al^3+^ ions: K^+^ should prefer Al^3+^ neighbors vs. Si^4+^ neighbors because of reduced electrostatic repulsion. This picture is confirmed by the DFT calculations and MD simulations discussed below.

The DFT calculations were performed with the Vienna Ab-initio Simulations Package (VASP)^46,47^ using the r^2^SCAN metaGGA^48^ exchange-correlation functional (see Methods for computational details). This functional describes well the structural properties of bulk mica (lattice constants and angles deviate less than 0.6% from the experimental values) and yields an improved value of the bulk modulus (54 GPa) compared to the GGA (PBE) functional used in previous studies^49,50^. Several models were tested, each characterized by different arrangements of K^+^ ions at the surface or Al^3+^ ions in the upper AlSi_3_ tetrahedral sheet (see Fig. 3b–e for a selection, and Supplementary Fig. 1 for the complete set). The K^+^ ions were placed in straight or zigzag rows, reproducing the preferred distributions observed experimentally (Fig. 3a). Each structure is further characterized by the arrangement of the substitutional Al^3+^ ions in the subsurface rings (meta or para), and, additionally, by their number and positions in the rings occupied by a K^+^ ion (see insets). The relative energies per K^+^ ion referred to the lowest-energy structure are shown at the bottom of each panel. Two main observations emerge from DFT: (i) The most stable K^+^ rows and K^+^ zigzag arrangements (Figs. 3b, d, respectively) are comparable in energy. Their small energy difference (Δ*E* ≈ 60 meV) is consistent with the coexistence of 120° kinks and straight rows observed in the experiments. (ii) Given a particular K^+^ order, structures with 2 Al per K-occupied ring are always favored compared to those with only 1 Al. This is exemplified by the comparison between Figs. 3b, c (Δ*E* ≈ 170 meV) and between Figs. 3d, e (Δ*E* ≈ 260 meV). The latter observation hints at a strong link between K^+^ and Al^3+^ order, further supported by the MC simulations below.

DFT was further employed to calculate hopping barriers for the K^+^ ions. The lowest calculated hopping barriers corresponded to K^+^ ions jumping from 1-Al- to 2-Al-rings, i.e., towards lower-energy configurations. This justifies the assumption of the MC simulations that only consider diffusion towards lower-energy states (see below). The calculated diffusion barriers lie between 0.7 and 1 eV, corresponding to a time scale for hopping between less than a minute and a few hours at room temperature. Hence, at least some diffusion events should be allowed during the ≈3 min passing between room-temperature cleaving and sample transfer into the AFM cryostat held at 4.7 K. This implies that the observed K^+^ distribution may not be uniquely determined by the interaction of the K^+^ ions with the tetrahedral sheets above and below them before cleaving; if diffusion is allowed, the system can approach the minimum-energy configuration dictated only by the lower sheet after cleaving.

**Fig. 4:**
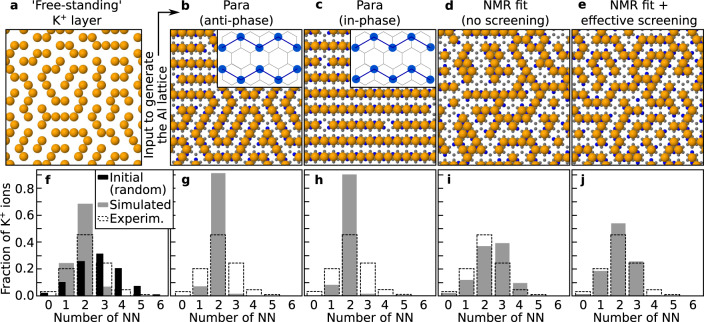
Monte Carlo simulations of K+ arrangements for different Al3+ arrangements. **a** Distribution of free-standing K^+^ ions (orange). **b**−**e** Distributions of K^+^ ions over different AlSi_3_ lattices (Al^3+^ and Si^4+^ ions are shown in blue and gray, respectively). **b**, **c** Long-range-ordered distributions of Al ions obtained assuming perfectly ordered para-only configurations. **d** Al distribution fitting the NMR data satisfying electrostatic constraints on the Al positions (1 or 2 Al/ring, no Al nearest neighbors (NN), meta and para equally favored)^52^. **e** Same Al distribution as in panel (**d**) but including screening effects through the substrate by considering a lower effective Al charge (details in Supplementary Note 2). **f**−**j** Histograms showing the fraction of K^+^ ions found with a given numbers of NN. The distribution extracted from the experimental data (dashed) is overlaid to the simulated ones. The best fit (**j**) is obtained for the K^+^ distribution in panel (**e**).

The link between the Al^3+^ and K^+^ order demonstrated by the DFT data was further explored by performing MC simulations (see Methods for the simulation details). The results are summarized in Fig. 4. The top row of Fig. 4 reports the MC-derived distributions of K^+^ ions. The bottom row shows the corresponding histograms of the NN distributions (gray), always overlayed on the distribution obtained from the experimental data (dashed). Figure 4a shows the distribution obtained for an unsupported K^+^ layer where the K^+^ positions were distributed on a lattice with the same lattice constant as mica. In this simulation, only the K^+^–K^+^ electrostatic repulsion can affect the K^+^ order. To obtain this distribution, the ions were initially randomly placed on the hexagonal lattice with 48% occupancy (the experimental concentration). Figure 4f shows the histogram of the initial (random) distribution, with a maximum at 3 NN (black). Relaxing the system by MC produces a pronounced maximum at 2 NN due to the frequent alternating rows seen in Fig. 4a (within each row, each ion has 2 NN; this distribution is favored because it minimizes the number of NN ions, and, hence, the electrostatic repulsion). The experimental distribution also has a maximum at 2 NN. However, this maximum is less pronounced, indicating a somewhat lower degree of order. The difference in the experimental K^+^ distribution compared to the simulated free-standing K^+^ layer is consistent with the K^+^ order being not uniquely determined by the K^+^−K^+^ interaction but also by the interaction with the underlying Al^3+^ lattice, as indicated by DFT.

It is here useful to open a parenthesis on the knowledge available from the literature on the Al^3+^ arrangement in the tetrahedral AlSi_3_ layers of mica. Direct measurements are currently missing. X-ray diffraction data indicate the lack of long-range order but cannot and do not yield information about the actual arrangements^51^. ^29^Si magic angle spinning (MAS)–NMR spectroscopy yields the probabilities of finding Si atoms in different tetrahedral environments (e.g., surrounded by 0, 1, or 2 Al atoms)^52^. Like the diffraction data, they do not allow on their own to determine the exact Al position in the rings (e.g., meta or para, when having 2 Al/ring) or the possible presence of short-range ordering. Previous MC simulations have determined possible distributions fitting the NMR data while obeying electrostatic constraints on the Al positions, specifically: Avoiding NN (Löwenstein’s rule)^53^ and having either 1 or 2 Al ions per ring to achieve maximum charge dispersion^36,52^. Among the tested distributions satisfying these constraints, the one best fitting the NMR data (in the following, referred to as the NMR fit distribution) had a 2:1 ratio of rings with meta and para configuration, consistent with meta and para being equally favored.

In the present work, MC simulations were performed to gain more information on the Al^3+^ order based on the measured surface K^+^ order (selected models in Fig. 4b–d; full set of simulated structures in Supplementary Fig. 2). The simulations were obtained starting from different subsurface Al^3+^ models, including some incompatible with the NMR data^52^—such as the long-range-ordered and random distributions in Fig. 4b, c and Supplementary Fig. 2d—and the NMR fit distribution mentioned above (Fig. 4d). The simulations evidence one common trait, consistent with the DFT data: The K ions always follow the order of the underlying Al lattice, preferably occupying rings with 2 Al ions. As expected, the NMR-incompatible Al distributions (e.g., Fig. 4b, c) yield K^+^ distributions that do not reflect the experimental data. More interesting, also the NMR fit yields an unsatisfactory agreement: In Fig. 4i, the simulated K^+^ distribution has too few K^+^ ions with one and too many with three neighbors compared to the experiments. However, one must notice that the models considered up to now do not consider any screening effects through the substrate. In reality, screening effects are to be expected: When considering only NN interactions, analysis of the DFT results indicates that the K–K and the K–Al interactions are comparable in energy (≈0.15 eV and 0.17 eV, respectively), despite the larger distance of K–K neighbors (0.52 nm) compared with K–Al (0.36 nm). Hence, the screened K–Al interaction should be described well by a weaker charge of Al. When taking a lower effective charge of Al as an approximation for screening (−0.65*e*, instead of −1*e*), one can obtain a good fit with the experimentally observed K arrangements (Fig. 4e, j). The extreme situation, when almost all of the Al formal charge is screened by the substrate (imposed charge on Al = −0.24*e*) yields a similar distribution as the “free-standing” K^+^ layer in Fig. 4a (Supplementary Figs. 2k, n). It is important to point out that the solution of Fig. 4e is not unique. Different ordering of the Al^3+^ sublattice than assumed here could yield the same ordering of the K^+^ ions and still be consistent with the NMR data (see Supplementary Note 2). Summarizing, the MC simulations confirm the important role of the subsurface Al^3+^ ions for the K^+^ order. They allow excluding many types of subsurface Al^3+^ arrangements, but even when combined with the experimental data on the K^+^ order, they do not yield a unique solution.

The short-range order reported here for the surface K^+^ ions of UHV-cleaved mica furthers the current knowledge about the mica surface. To the authors’ knowledge, the intrinsic surface K^+^ order has not been determined directly in the previous literature. Early LEED works did not provide any evidence of ordering after UHV cleaving^39^, in contrast with the short-range order reported here. In the present work, LEED could not be performed on the freshly cleaved samples because of charging. Patterns compatible with those in ref. 39 could be acquired on contaminated samples (see Supplementary Note 6). LEED is also unlikely to observe much short-range ordering on a lateral scale smaller than a few nanometers^45^. Contrasting to the scarcity of UHV studies, more evidence about ion ordering on mica is found for surfaces in solution. AFM studies have shown that hydrated K^+^ and Rb^+^ ions^25,54^ stabilized at mica-solution interfaces exhibit a similar ordering to that of the K^+^ ions on the freshly cleaved surface reported here, i.e., preferably arranged in row segments rather than randomly. With support from molecular dynamics simulations, it was concluded^24^ that the ordering was related to water-induced correlation effects, i.e., the energy gained by the global hydration structure when having a specific geometric arrangement of the ions, rather than to repulsion between the solvated ions or electrostatic interaction with the substrate. However, the similarity of the arrangements found in solution to those of the UHV-cleaved surface suggests that the interaction between ions and substrate may play a more significant role than previously assumed in determining the arrangement of (hydrated) ions on mica.

In conclusion, this study provides atomic resolution on the surface details of mica, a popular layered mineral in surface and interfacial science. Atomically resolved nc-AFM images after UHV cleaving reveal the intrinsic short-range ordering of its surface K^+^ ions: These preferentially arrange in short, alternating rows, in contrast to previous assumptions of random arrangements. DFT calculations and MC simulations show that the K^+^ ordering is not only due to the electrostatic repulsion between the K^+^ ions but also strongly affected by the interaction with Al^3+^ ions in the subsurface aluminosilicate layer. The atomic-scale insights provided by this study on UHV-cleaved mica broaden the current knowledge of mineral surfaces. They also offer valuable input to disentangle the many factors at play in the more complex ambient or liquid environments, where mica serves as a model system to unravel important atomic-scale processes.

## Methods

### Experimental methods

The experiments were carried out in a UHV setup consisting of two interconnected chambers: a preparation chamber for sample cleaving and XPS measurements (base pressure <1 × 10^−10^ mbar) and an AFM chamber for nc-AFM measurements (base pressure <2 × 10^−11^ mbar).

Natural muscovite mica single crystals [(0001) oriented disks of grade V1, with 10 mm diameter and 0.25 mm thickness, from TedPella—see Supplementary Fig. 5 for typical impurities] were glued on Omicron-style stainless steel sample plates with UHV-compatible epoxy glue (Epotek). They were cleaved in UHV at room temperature before each experiment. Two cleaving methods were used. The first consists of using a wobble stick to apply a tangential force to a metal stud glued on top of the sample^55^. The portion of the sample initially covered by the stud is thus cleaved, and it can be probed with nc-AFM. The second method uses a carbon-steel blade mounted on an Omicron-style plate to peel off a thin mica layer. This procedure induces the cleavage of an entire mica disk and can be repeated on a single sample several times. The latter technique was used for the presented XPS experiments. AFM data on the as-cleaved surfaces were acquired with both methods and yield identical surface structures.

XPS was performed with a non-monochromatic dual-anode Mg/Al X-ray source (SPECS XR 50) and a hemispherical analyzer (SPECS Phoibos 100). Spectra were acquired in normal and grazing emission (70° from the surface normal). Due to the insulating nature of the samples (bandgap of 7.85 eV)^56^, the XPS spectra showed shifts to higher binding energies (between 5 and 7 eV). As already noted in the literature^35^, the magnitude of the shift depends on the amount and type of surface contamination, XPS acquisition geometry, and sample thickness. For the display and analysis of the XPS data, an energy correction was applied to all spectra to set the peak value of the core-level K 2*p*_3/2_ to 293.75 eV reported in the literature^35^. This resulted in an O 1 *s* peak at binding energy of 532.3 eV. The intensities and positions of the Al-Kα-excited XPS peaks were evaluated with CasaXPS after subtracting a Shirley-type background. The K 2*p* peaks were fit by two doublets, assuming an asymmetric Lorentzian line shape LA(1, 643). In each doublet, the relative peak separation was set to 2.8 eV in line with previous works^35^, and the area ratio to 2:1. All peaks have the same FWHM. The separation between the two doublets was set the same for normal and grazing emission.

The AFM measurements were performed at 4.7 K using a commercial Omicron qPlus LT head and a differential cryogenic amplifier^57^. Frequency-modulated non-contact AFM mode was used. The tuning-fork-based AFM sensors (*k* = 2000−3500 N/m, *f*_0_ ≈ 45 kHz, Q ≈ 50,000)^40^ had a separate contact for tunneling current attached to electrochemically etched W tips that were cleaned in situ by field emission^58^. Before each measurement, the tips were further prepared on a clean Cu(110) single crystal by repeated indentation and voltage pulses. CO-functionalized tips^59^ were used to image some samples. The coarse approach was done with a setpoint of −1 Hz. The controller was switched off, and the tip was gradually approached in constant-height mode until an AFM contrast was visible while scanning in x and y. All AFM images presented here were acquired in constant-height mode. At times, the absolute values of frequency shifts obtained during the acquisition of atomically resolved images were large (up to 100 Hz) and were not reproducible on different regions on the same sample or on different samples. This is because cleaving can create domains of trapped charges that can cause long-range electrostatic interactions between the surface and the tip^38^. Short-range forces used for imaging the K^+^ ions were in the order of 0.1 nN (attractive regime). The electrostatic fields created after cleaving can be partially compensated by applying a bias voltage between tip and sample. Most of the measurements were performed by applying a bias voltage such that the surface was measured as close as possible to the lowest local contact potential difference (LCPD), i.e., at tip-sample potential differences that were as close to the LCPD as possible. The bias voltage reported in the presented images corresponds to bias applied to the back of the sample plate while having the tip on ground. Supplementary Note 5 reports details about the statistical analysis of AFM images.

### Computational methods (DFT)

The DFT calculations were performed with the Vienna Ab-Initio Simulation Package (VASP)^46,47^ using projector augmented wave (PAW) potentials^60^ and the r^2^SCAN metaGGA^48^ exchange-correlation functional. The bulk structure was optimized with a cutoff energy of 1000 eV, and a k-point mesh of 4 × 2 × 1 was used to integrate the Brillouin zone. The optimized r^2^SCAN lattice parameters (*a* = 5.16 Å, *b* = 9.00 Å, *c* = 20.19 Å, and β = 95.75°) are in excellent agreement with the experimental values (*a* = 5.16 Å, *b* = 8.95 Å, *c* = 20.07 Å, and β = 95.99°)^61^ (maximal deviation of ~0.6%).

The surface calculations were performed with a lower cutoff energy of 500 eV and a 2 × 2 × 1 k-point mesh for the 2 × 1 unit cell (see Fig. 3). A single muscovite trilayer was used for the calculations, as double trilayer slabs yielded differences <10 meV/K^+^ ion. The rows were formed along the [100] direction. Their rotation by 60° (tested because the surface does not possess an exact hexagonal symmetry) gave similar results, with differences <15 meV/K^+^ ion. A symmetric setup was used for the distribution of the Al ions on both sides of the slab.

The diffusion barriers of the K^+^ ions were determined by identifying the saddle points in the potential energy landscape using the improved dimer method^62^, with the remaining forces at the saddle point smaller than 0.02 eV/Å. This was followed by the relaxation of the K^+^ ion from the saddle point to either the initial or the final state of the reaction step. Core-level shifts were determined in the initial state approximation.

### Simulation methods (Monte Carlo simulations)

Electrostatic models consisting of a K layer sitting above an AlSi_3_ layer were built inspired by the Metropolis algorithm^63^. Al ions were constrained to occupy ¼ of the lattice positions in the hexagonal rings of the AlSi_3_ tetrahedral sheet. K ions were constrained to sit on a hexagonal lattice centered in the hexagonal rings of the AlSi_3_ tetrahedral sheet, at a vertical distance of 1.85 Å from the underlying layer, according to the values derived from the DFT-optimized surface (see Supplementary Table 1). Unless noted otherwise, screening effects via the substrate were not accounted for.

To build the model, the subsurface AlSi_3_ layer was generated within a circular area with a radius of ≈100 nm (Al ions treated as −1 point charges, Si ions as neutral). The full set of simulations in Supplementary Fig. 2 was obtained with different arrangements for the Al sites, as discussed in Supplementary Note 2. As a second step, the surface layer of K ions (+1 point charges) was added by randomly placing the charges on the same 100-nm-radius area of the AlSi_3_ layer. A coverage of 48% was chosen to match the experimental findings. During the simulations, ions in a central area of ≈30 × 30 nm^2^ above the subsurface area were allowed to move. The rest was frozen to avoid electrostatic expansion. After pre-calculating the field of the immobile ions, the simulation was run according to the following algorithm: Make a random jump of a randomly selected mobile K ion to an unoccupied neighboring site and accept the new configuration if its electrostatic energy has decreased. This procedure is motivated by the DFT-calculated barriers, which only allow energetically downhill diffusion at room temperature (see main text). The steps were repeated until reaching a local energy minimum, in which no successful jump appeared during 100 attempts per atom. The minimum was typically reached after 1.6–1.9 successful jumps per ion.

To compare the ion distributions in different models with the experimental distributions, histograms of the fraction of K^+^ ions with a given number of nearest neighbors (NN) were taken as a metric (see, e.g., Fig. 4f–j). They represent the number of ions found with a given number of NN. Histograms of the experimental distributions were obtained from a point map of the ion positions, extracted as detailed in Supplementary Note 5.

## Supplementary information

Supplementary Information

## Data Availability

The data that support the findings of this study are available from the corresponding author upon request.
