# Supplementary material for: Resolving the intrinsic short-range ordering of K+ ions on cleaved muscovite mica
Source: Nat Commun. 2023 Jan 13;14:208. doi: 10.1038/s41467-023-35872-y (PMC9839703; doi:10.1038/s41467-023-35872-y)
Supplement: Supplementary file 1 — Supplementary Information [file 41467_2023_35872_MOESM1_ESM.pdf]

Supplementary Information – Resolving the intrinsic short-range ordering of K^+ ions on cleaved muscovite mica

G. Franceschi et al.*

*franceschi@iap.tuwien.ac.at

Supplementary Figures……………………………………………………..…Page 2

- Supplementary Figure 1: Distributions of surface K^+ and subsurface Al^{3+} ions: full set of structures calculated by DFT
- Supplementary Fig. 2: Full set of Monte Carlo simulations of K distributions for different Al distributions
- Supplementary Fig. 3: MC-simulated relaxation of the K ion morphology with the best fit to the experimental data (Fig. 4e in the main text)
- Supplementary Fig. 4: Structural elements for the Al arrangement in the $AlSi_3$ layer
- Supplementary Fig. 5: XPS on the UHV-cleaved surface
- Supplementary Fig. 6: AFM spectroscopies
- Supplementary Fig. 7: Statistical analysis of the nc-AFM images
- Supplementary Fig. 8: Comment on Fourier transforms

Supplementary Tables…………………………………………………...….Page 8

- Supplementary Table 1

Supplementary Notes…………………………………..…………...….Page 9

- Supplementary Note 1: Additional details on the Density Functional Theory (DFT) calculations
- Supplementary Note 2: Additional details on the Monte Carlo simulations
- Supplementary Note 3: XPS survey spectrum and C 1s region of UHV-cleaved mica
- Supplementary Note 4: AFM-based spectroscopies
- Supplementary Note 5: Analysis of the nc-AFM results
- Supplementary Note 6: Comment on LEED

Supplementary References…………………………..……..………...….Page 17

Supplementary Figures

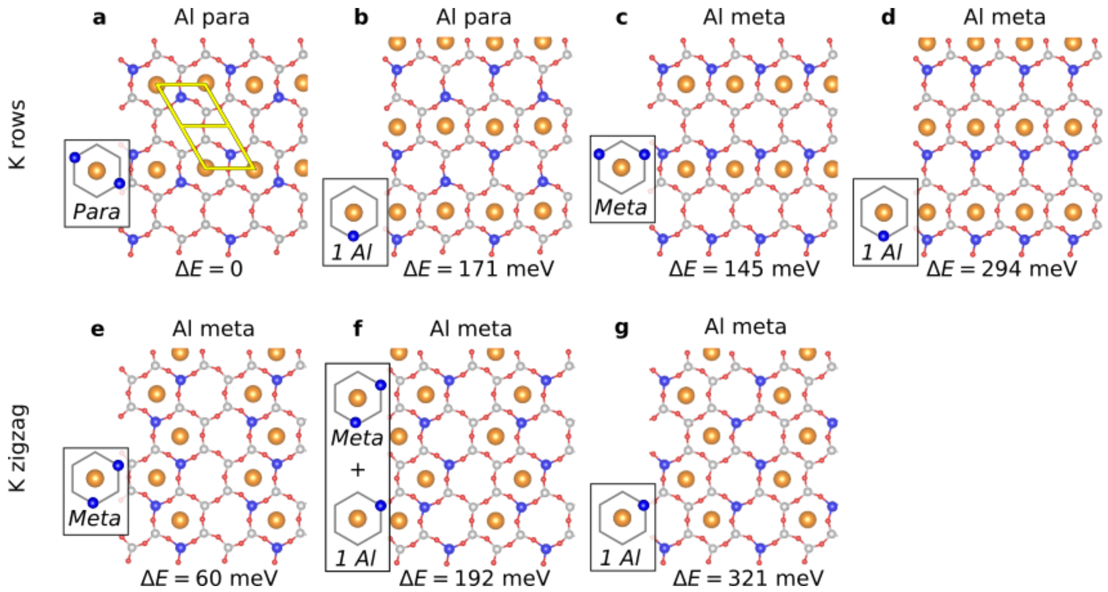

Supplementary Fig. 1. Distributions of surface K^+ and subsurface Al^{3+} ions: full set of structures calculated by DFT. (a–g) Structures (top view) and energy differences per K^+ ion with respect to the lowest-energy structure in panel **a**. Each structure is identified by the K order (rows or zigzag, in the upper and lower row, respectively), the Al order in the substrate (meta or para), and by the position and number of the Al ions in the K-occupied rings (1 or 2, the latter in para or meta configuration – see corresponding insets).

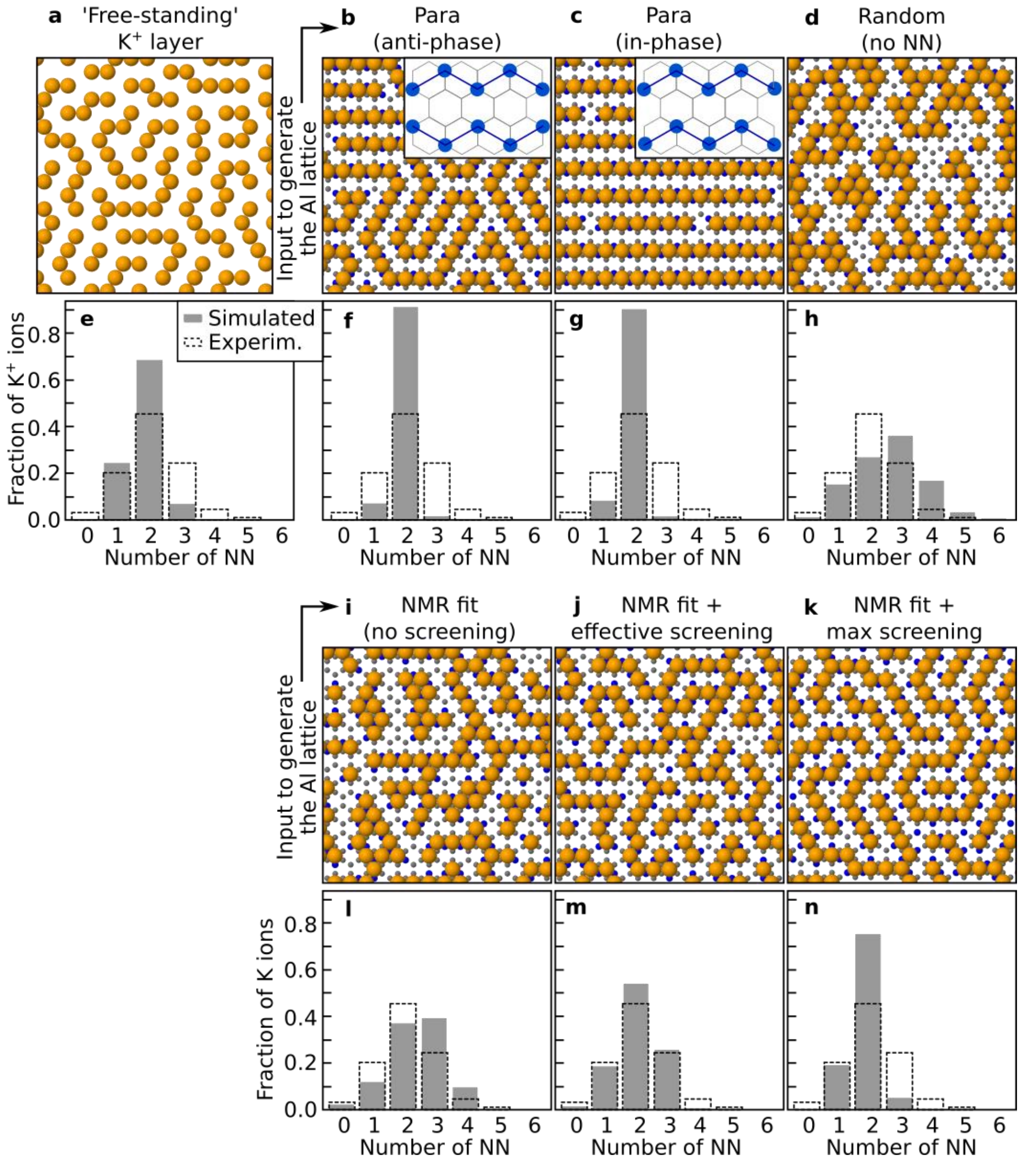

Supplementary Fig. 2. Full set of Monte Carlo simulations of K distributions for different Al distributions. **a−d**, **i** Distributions of K^+ ions (orange) obtained over $AlSi_3$ lattices (Si: grey, Al: blue) in a purely electrostatic model that considers K and Al as +1 and −1 point charges. **i−k** Distribution of K^+ ions over an $AlSi_3$ lattice fitting the NMR data[1] (see main text) and accounting for different screening effects through the substrate (different effective charge of Al, q_{Al}): **i** no screening, $|q_{Al}| = 1e$; **j** medium screening, $|q_{Al}| = 0.65e$; **k** strong screening ($|q_{Al}| = 0.24e$) yielding almost the same distribution as the free-standing layer of panel **a**. **e−h, l−n** Histograms representing the fraction of K^+ ions found with given numbers of nearest neighbors (NN). The grey bars show the simulation results. Dashed bars correspond to the distributions extracted from the experimental data.

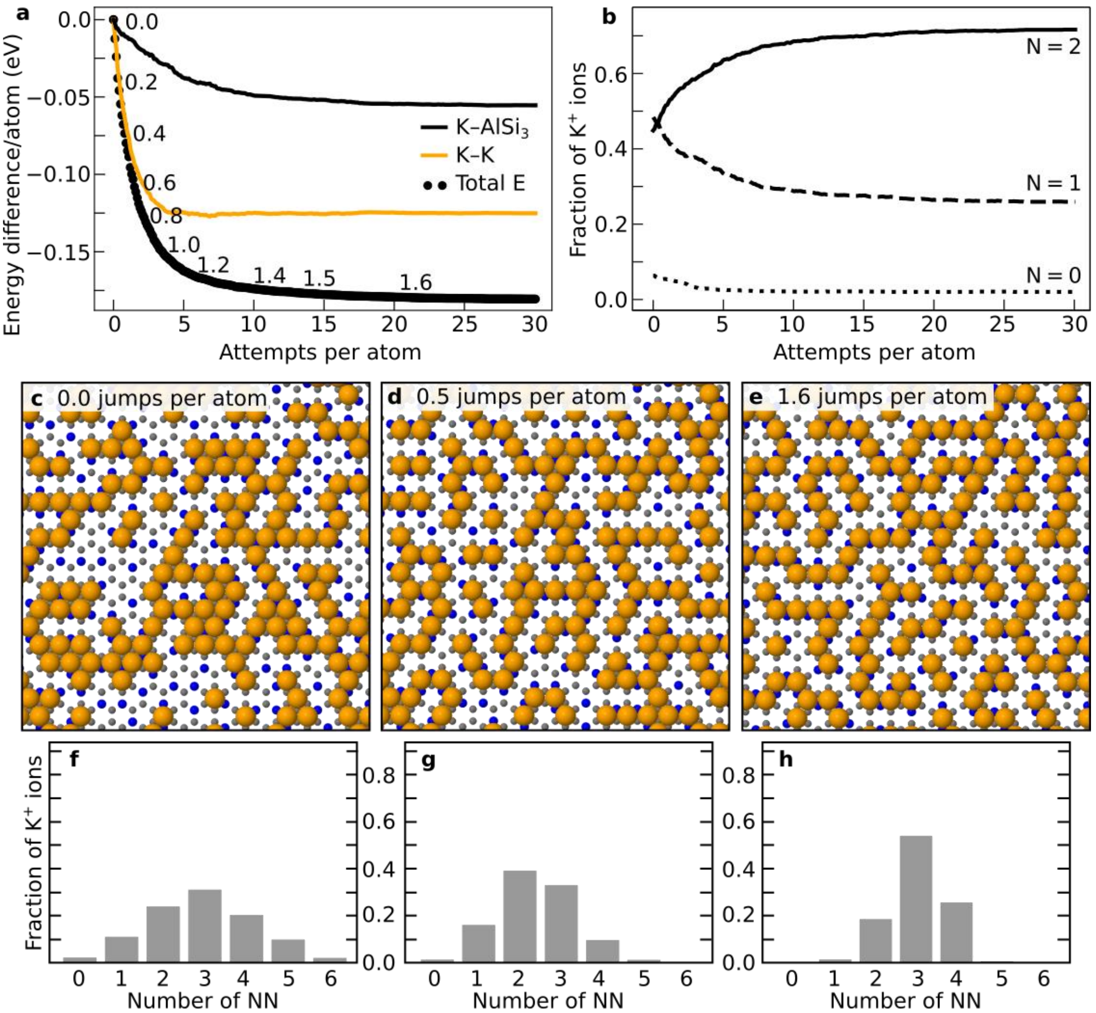

Supplementary Fig. 3. MC-simulated relaxation of the K ion morphology with the best fit to the experimental data (Fig. 4e in the main text). a Evolution of the total electrostatic energy and its components. The numbers indicate the realized hops per atoms. The energy values are referred to the initial state. **b** Evolution of the relative numbers of K ions in rings with $N = 0$, $N = 1$, and $N = 2$ Al atoms. **c−e** Snapshots of the morphology at different numbers of attempts per atom. **f−h** Histograms showing the fraction of K^+ ions found with a given number of NN for the configurations in panels **c−e**.

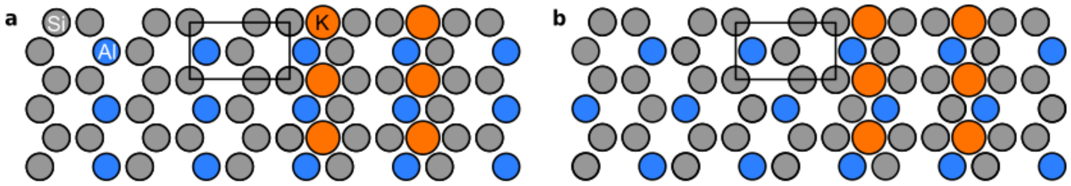

c

	2Al	1Al	0Al
NMR	0.12	0.60	0.28
(a)	0.33	0.33	0.33
(b)	0.00	1.00	0.00

Supplementary Fig. 4. Structural elements for the Al arrangement in the $AlSi_3$ layer. a, b Fully ordered $AlSi_3$ layers, with Al in only meta, and only para configurations in the rings with 2 Al, respectively. **c** Experimental and calculated probabilities of finding Si atoms with 2, 1, and 0 Al NN. An appropriate mixture of models **a** and **b** can yield the NMR-derived probabilities when accounting for the deviation from perfect 1:3 Al:Si stoichiometry in the tetrahedral sheets. Due to the preference of K for maximizing the number of Al neighbors, the minimum-energy K configuration for these structures would correspond to perfectly ordered K rows.

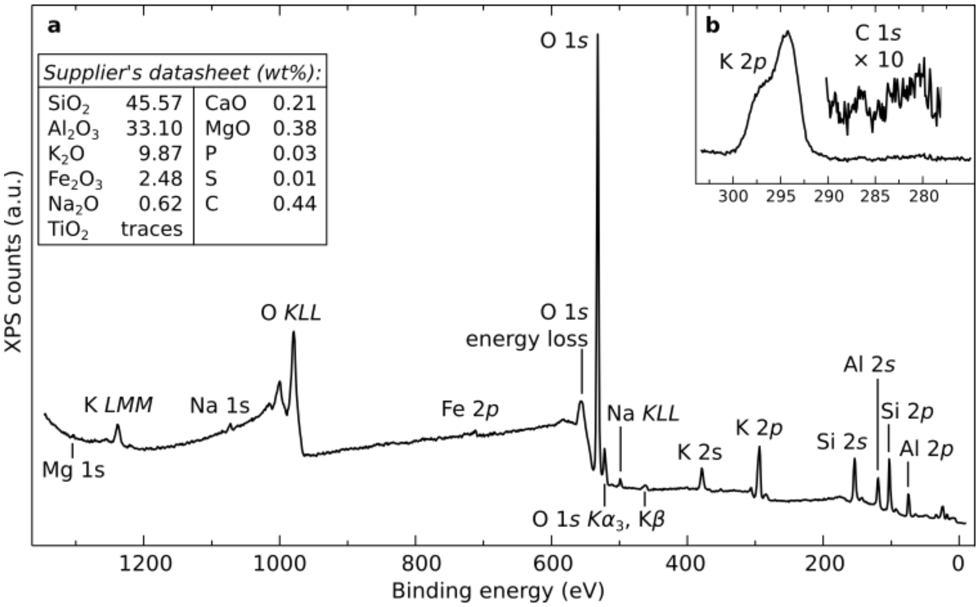

Supplementary Fig. 5. XPS on the UHV-cleaved surface. a, b XPS survey and K 2*p*+C 1*s* region (after removing the Al Kα_3 satellite) of an as-cleaved mica surface (Al Kα, 1486.61 eV, 70° grazing emission, pass energy 60 eV, and 20 eV, respectively). The inset reports the chemical composition from the supplier[2]. The binding energy axes were adjusted to account for charging (see Methods).

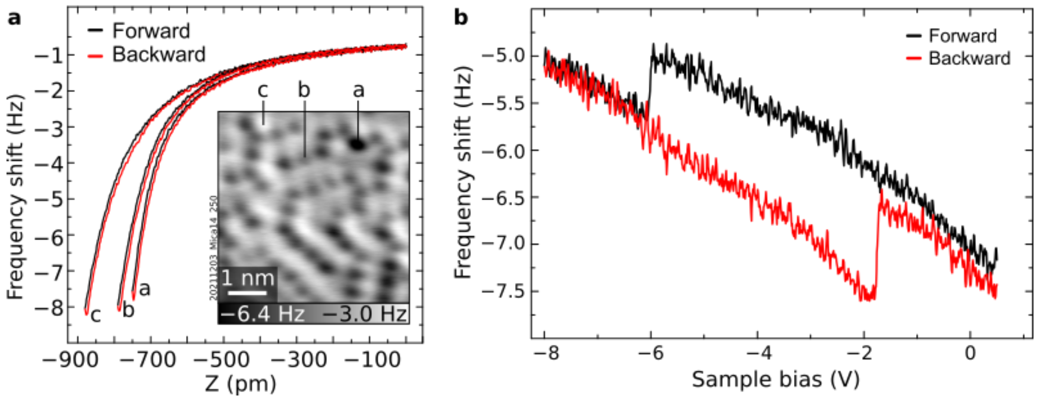

Supplementary Fig. 6. AFM spectroscopies. **a** Frequency shift vs. tip-sample distance acquired with a CO tip on three types of features on the cleaved mica surface labeled as “a”, “b”, and “c”. Each curve averages over three runs on the same atom (forward: black, backward: red). $V_s = -10$ V. **b** Typical jumps in the frequency shift during the acquisition of a Kelvin parabola on an as-cleaved mica surface.

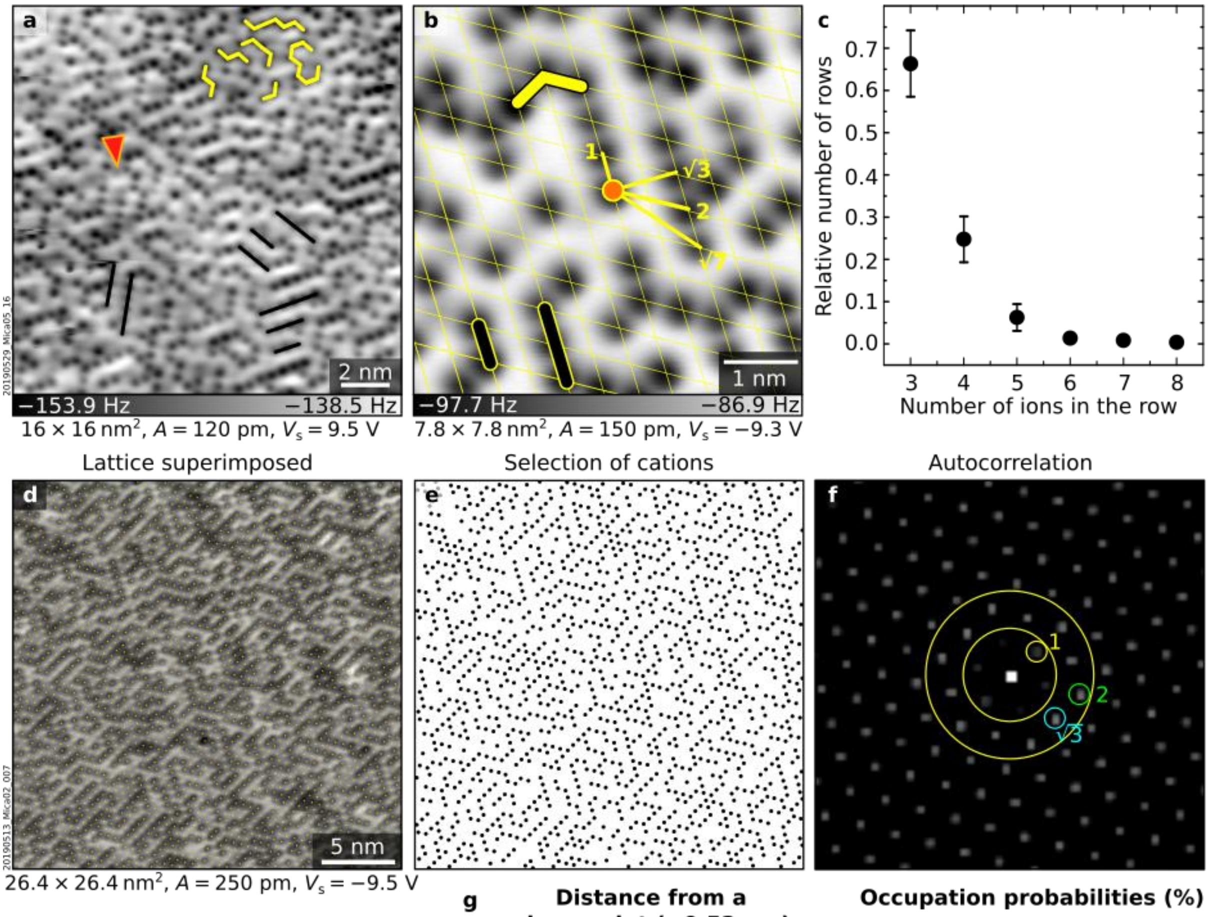

g

Distance from a given point (×0.52 nm)	Occupation probabilities (%)	
	Experiments	Simulations
1	35.4 ± 1.3	34.8 ± 1.7
2	50.1 ± 0.8	52.0 ± 1.4
√3	49.6 ± 1.1	50.2 ± 0.6
√7	45.6 ± 0.7	46.4 ± 1.0
average	47.8 ± 0.1	48.0

Supplementary Fig. 7. Statistical analysis of the nc-AFM images. a, b nc-AFM images highlighting **a** common ion arrangements, i.e., alternating rows and 120° kinks (black and yellow, respectively), and **b** the hexagonal lattice of mica (yellow) with selected ion-ion distances d in units of 0.52 nm. The red arrow in **a** points to a species of fainter contrast, assigned to a Na^+ ion. **c** Normalized number of straight ion rows or row sections made of a given number of ions. **d−g** Statistical analysis of the ion positions on the as-cleaved mica surface. **d** nc-AFM image of a UHV-cleaved mica surface with the hexagonal lattice derived from its Fourier transform superimposed (yellow). **e** Lattice of the K^+ ions identified with a threshold approach (not accounting for the faint species). **f** Autocorrelation of **e**. Yellow circles aid the eye in identifying regions of different occupation probability (brighter contrasts correspond to higher probabilities). Selected points at $d = 1$, 2, and √3 from the reference ion are marked. **g** Occupation probabilities as a function of distance extracted from the experimental data and from the MC simulation of Fig 4e.

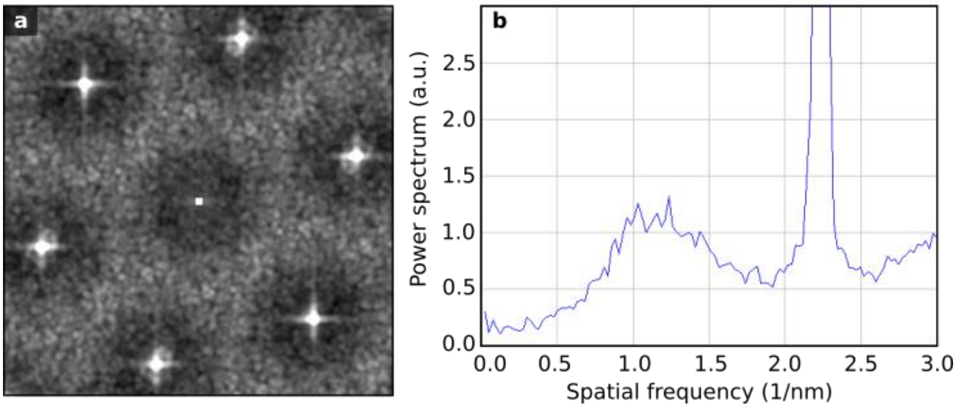

Supplementary Fig. 8. Fourier transform of the experimental potassium ion distribution. **a** Fourier transform of the mask in Supplementary Fig. 6e. The crosses at the strongly overexposed first-order maxima are artifacts caused by the input image being a square. **b** Plot of the diffuse intensity of the power spectrum as a function of the spatial frequency, obtained by averaging over all directions in the power spectrum.

Supplementary Tables

Supplementary Table 1: Calculated relative heights and projected total charges for K^+ ions occupying aluminosilicate rings characterized by 0, 1, 2, or 3 Al atoms.

# of Al atoms in the occupied ring	Relative height (Å)	Projected total charge (*e*)
0	1.84	8.01
1	1.89	8.03
2	1.84	8.03
3	1.97	8.05

Supplementary Notes

Supplementary Note 1: Additional details on the Density Functional Theory (DFT) calculations

Full set of tested DFT structures

Supplementary Figure 1 shows the complete set of DFT-tested structures, highlighting the order of the K and Al ions, the arrangement of the Al ions in the K-occupied rings, and the energy per K^+ ion referred to the lowest-energy structure of Supplementary Fig. 1a.

Relative height and projected total charge for K^+ ions in 0, 1, 2, 3 Al rings

Supplementary Table 1 summarizes the relative heights and projected total charges of K^+ ions positioned in different aluminosilicate rings. The values were calculated after fully relaxing the ionic positions in a single trilayer slab. They were almost identical for a double trilayer slab (differences <0.01 Å and <0.01*e* for the ionic positions and the projected total charges, respectively).

The relative heights were calculated as the difference between the z coordinate of the surface K^+ ion and the average of the z coordinates in the $AlSi_3$ subsurface plane. All values are very similar, except for the case of 3 Al atoms in the occupied ring. This arrangement is considered a defect, since it does not comply with the principle of maximum charge dispersion[1]. Thereby, the predicted height difference of ≈0.1 Å compared to the non-defective rings is expected to yield a visible difference in the nc-AFM images. It is possible that the very dark species sparsely seen in the images correspond to K^+ ions sitting on these sites. Another possibility is that these darker species correspond to substitutional Ca^{2+} species (see Supplementary Note 5 below).

The projected total charges were calculated as the sum of the projected partial charges of the *s*, *p*, and *d* orbitals within the PAW sphere, setting LORBIT=11 (VASP version 6.3.0). The projected total charge for a K ion in the bulk is equal to 8.00*e*, similar to the values for surface K ions in Supplementary Table 1.

Supplementary Note 2: Additional details on the Monte Carlo simulations

As mentioned in the Methods Section, the MC simulations shown in this work stem from a simplified model that only considers atom-atom electrostatic interactions with a 1/r potential. Other factors that might play a role, e.g., covalent bonding contributions and variations of

lattice relaxations at the K sites, have not been included for the following reasons: The influence of covalent bonding contributions should be limited, as the bonding of K is expected to be mostly ionic and the variation of the covalent bonding strength among different K sites should be small. Also lattice relaxations, both out-of-plane and in-plane, should not be critical; simulations assuming the bulk interlayer distance of 2.28 Å between the K layer and the tetrahedra sheet (not shown) are very similar to the simulations in Fig. 4 and Supplementary Fig. 2. There, the distance obtained from the DFT-relaxed cleaved surface (1.85 Å) was assumed. In the DFT-simulated structures, the in-plane displacements of the K ions compared to the ideal lattice sites is less than 4% of the K–K NN distance. This suggests that lattice relaxations should not play a significant role for the arrangement of the K^+ ions. The largest error of the simulation energies probably comes from approximating the screening of the electrostatic interactions by the substrate, approximated by a reduced effective charge on the Al. This neglects the distance-dependence of the screening, and the screening of the K–K interactions. Developing a high-accuracy screening model would be difficult, as some of the required parameters – e.g., the layer-resolved anisotropic dielectric constant at the timescale of single diffusion events – are not known. In any case, the simple model used here should be sufficient, as the simulations are mainly determined by the strong interactions of nearby ions and insensitive to quantitative values: They only depend on whether a diffusion step is uphill or downhill in energy.

Supplementary Figure 2 shows the complete set of MC-simulated structures, i.e., the results presented in Fig. 4 plus additional simulations. The simulations were obtained with different distributions of the Al atoms in the subsurface $AlSi_3$ sheet. Supplementary Figures 2b, c show long-range ordering according to the corresponding insets. Supplementary Figure 2d is based on an Al distribution with no nearest neighbors (NN), according to the known Löwenstein's rule for aluminosilicates[3], a maximum of 2 Al ions/ring, but otherwise a random arrangement. Supplementary Figure 2i is based on an Al arrangement compatible with the NMR data in ref. [1] and satisfying electrostatic constraints on the Al positions, i.e., no NN, maximum 1 or 2 Al/ring, and random placement apart from these constraints. The meta:para ratio obtained with these constrains corresponds to 2:1[1]. Supplementary Figures 2j, k are based on the same Al distribution of Supplementary Fig. 2i but include an approximation for screening effects through the substrate to depict a more realistic scenario. The effect of screening is simulated by reducing the formal charge of the Al ions and spreading the missing charge over the Si ions in the $AlSi_3$ layer to retain a charge-neutral system. Considering a weaker charge on the Al sites is reasonable: When considering only NN interactions, DFT

indicates that the K−K and the K−Al interactions are comparable in energy (≈0.15 eV and 0.17 eV, respectively), despite the larger distance of K−K neighbors (0.52 nm) compared with K−Al (0.36 nm). Hence, the K−Al interaction should be described well by a weaker charge of Al. The effective charge of −0.65*e* chosen for the distributions in Supplementary Figs. 2j (reprinted in Fig. 4e of the main text) yields the best fit with the experimentally observed K arrangements.

Supplementary Figure 3 demonstrates the evolution of the K ion morphology during the simulation using the subsurface Al configuration compatible with NMR and the Al effective charge best fitting the experiment, i.e., the setup of Fig. 4e and Supplementary Fig. 2j. Supplementary Figure 3a shows the evolution of the total electrostatic energy of the configuration and its contributions. The most prominent contribution is the relaxation due to K–K repulsion (solid orange line), much stronger than the K–Al contribution (solid black line). The numbers in the graph indicate the average number of jumps per atom to reach a given energy. Supplementary Figures 3c−e depict the evolution of the morphology: Panel (c) shows the initial random configuration, while panels (d) and (e) correspond to the configurations after 0.5 and 1.8 realized jumps per atom, respectively. Supplementary Figures 3f−h show the corresponding histograms of NN K ions. Supplementary Figure 3b presents the evolution of the correlation between the K ions and the subsurface Al ions. It shows the relative numbers of K ions in rings containing $N = 0$, $N = 1$, and $N = 2$ Al atoms. A tendency to occupy rings with 2 Al ions is evident.

Comment on additional Al arrangements in the $AlSi_3$ layer

In this work, it was shown that the measured distribution of K^+ ions can be fitted well by considering the (screened) interaction with a subsurface $AlSi_3$ layer that satisfies electrostatic constraints on the Al positions and fits previous NMR data[1]. This model, referred to as NMR fit, and based on a statistical placement of the Al atoms with the posed constraints, is not unique, however. Here it is argued that other solutions with more pronounced ordering fit the NMR data; these may influence the surface K^+ ordering.

Consider the two fully ordered $AlSi_3$ layers in Supplementary Fig. 4, with Al ions in (a) meta and (b) para positions. An appropriate mixture of structures would fit the NMR data: The first row in panel (c) shows the experimental probabilities for Si ions in different environments, i.e., with 2 Al, 1 Al, and 0 Al NN. The underlying rows yield the probabilities derived from the fully ordered models in (a) and (b). Considering that the difference between the 2 Al and 0 Al values in NMR are only due to an Al deficiency (Al:Si concentrations in the tetrahedral sheet

< 1:3), a good match with experiments may be achieved by appropriately mixing the two structures. The structures in Supplementary Figs. 4a, b would favor alternating K^+ rows. In other words, a mixture of these fully ordered models can be compatible with the NMR data and, at the same time, favor K^+ rows. Clearly, the fully ordered models considered here are extreme scenarios not occurring in reality. Nonetheless, this hypothetical structure indicates that structures with local ordering are compatible with the NMR data and, at the same time, favor short-range ordering of the K^+ ions exceeding that observed in the experiments.

Supplementary Note 3: XPS survey spectrum and C 1*s* region of UHV-cleaved mica

Supplementary Figure 5a shows an XPS survey acquired in grazing emission on a UHV-cleaved mica surface. The spectrum is consistent with others found in the literature[4,5], showing trace amounts of Mg, Fe, and Na besides the elements characteristic of mica (O, K, Si, and Al). The inset in panel (b) shows the K 2*p* peak and the adjacent C 1*s* region after removing the Al $K_{\alpha 3}$ satellite. The sample is virtually free from carbon.

Supplementary Note 4: AFM-based spectroscopies

Supplementary Figure 6 shows two AFM-based spectroscopies performed on UHV-cleaved mica surfaces: frequency shift vs. tip-sample distance and frequency shift vs. sample bias voltage (Supplementary Figs. 6a, b, respectively).

Supplementary Figure 6a plots curves acquired on representative features identified based on their contrast: Features labeled as “a” are the round species appearing darker than average K^+ ions. Black dots assigned to “regular” K^+ ions are labeled as “b”, and white regions in the background as “c”. Before each acquisition, the tip was positioned on the chosen feature and retracted by 500 pm from the acquisition height of the image in the inset. In Supplementary Fig. 6a, the same value of frequency shift occurs at different tip-sample distances for the three different features: The biggest tip displacement towards the sample is required in region “c” between the K^+ ions, followed by features “b” and then “a”. This indicates that darker features protrude more than brighter ones or that they attract the tip more strongly due to a higher local charge or different chemical contrast. Note that the ions were almost exclusively measured in the attractive regime regardless of the tip termination, even at extremely close distances. Hence, a minimum in the curves of Supplementary Fig. 6a could not be observed. The exclusively attractive interaction between isolated, undercoordinated cationic adatoms on the surface and

the tip is typical in nc-AFM[6,7]. Note also that the curves are quantitatively reproducible only within regions of a few nanometers that show the same background contrast. Quantitative values vary over distances of a few nanometers because the charges introduce a significant contribution of long-range forces to the overall tip-sample interaction.

$\Delta f(V)$ curves (Kelvin parabolas), used to calculate the local contact potential difference (LCPD), were acquired on features "a", "b", "c" of Supplementary Fig. 6a on different UHV-cleaved mica surfaces (before acquiring each curve, the tip was positioned on the chosen feature and retracted by 500 pm from the height at which the image in the inset was acquired). The general trend is that Kelvin parabolas acquired on more attractive features have their maxima shifted in sample bias by roughly −2 V compared to Kelvin parabolas taken on the background, indicating that the attractive features are positively charged, as expected for K^+. Unfortunately, quantitative conclusions cannot be drawn: Many curves appear like in Supplementary Fig. 6b, with one or two sudden jumps in the frequency shift. After such a jump, the Kelvin parabola acquired on the same position is shifted in bias compared to the previous one. The behavior is interpreted as charging/discharging events. Ion manipulation can be excluded since images acquired before and after the jump events show the same arrangement of the K ions. The smaller the tip-sample distance, the more probable the events. As for the $\Delta f(z)$ curves discussed above, quantitative reproducibility is hindered by the presence of regions with different long-range interactions with the tip.

Supplementary Note 5: Analysis of the nc-AFM results

Supplementary Figures 7a, b show nc-AFM images of UHV-cleaved mica while highlighting common ion arrangements on UHV-cleaved mica, i.e., alternating rows (black) and 120° kinks (yellow) that often interrupt or connect rows. Supplementary Figure 7b shows a detailed view of the mica structure, the hexagonal lattice (yellow), and some relevant distances d in units of 0.52 nm. The separation between the two ions next to a 120° kink is $d=\sqrt{3}$.

Counting K^+ ions

Supplementary Figures 7d–f illustrate the typical analysis performed via the software ImageJ[8] to count and evaluate the distribution of the K^+ ions on UHV-cleaved mica surfaces. The analysis was performed on ≈10 images from different samples with similar image quality to Supplementary Fig. 7a. Image sizes ranged from ≈15 × 15 nm^2 to ≈40 × 40 nm^2, corresponding to an absolute number of counted features per image between 600 and 3600. Each nc-AFM image was first undistorted based on its Fourier Transform[9]. The reference lattice obtained

from a back transform of the first-order Fourier spots was superimposed on the undistorted nc-AFM image, as shown in Supplementary Fig. 7d. In Supplementary Fig. 7d, all ions (black dots) sit on the reference lattice (yellow). The ions were selected and counted with a threshold approach, producing the mask shown in Supplementary Fig. 7e.

This analysis yielded a coverage of ≈46% when counting uniformly dark species, and 47.8±0.1% when selecting also fainter species such as the one highlighted by a red arrow in Supplementary Fig. 7a and Figs. 1c, d (errors in the statistical evaluations of AFM images represent 95% confidence intervals calculated with a two-tailed Student's t-distribution from the standard error of the mean). This value is smaller than the 50% expected for perfectly stoichiometric samples. The most likely reasons for this discrepancy are: (i) An Al:Si ratio higher than 1:3 in the tetrahedral sheets, which would require a lower K concentration in the K layer to achieve charge neutrality. Such a deviation from the ideal stoichiometry was already reported for the samples used observed by Herrero et al.[1] (3.16 and 0.84 for Si and Al in the tetrahedral sheet, and 0.84 in the K interlayer, which contained also trace amounts of Ca^{2+}) and is consistent with the datasheet of the samples used here (see Supplementary Fig. 5). (ii) Presence of +2 impurities replacing the surface K^+. In this case, fewer ions would be needed at the surface because the same negative charge can be compensated by half the number of +2 species compared to +1 species. These species could be Ca^{2+} ions, which are present according to the supplier's datasheet, and are supposed to substitute K^+. Because of their higher charge, these species should appear darker than K^+ ions (the average features) in nc-AFM. In fact, some species darker than average are recognized in the images (arrows highlight examples in Figs. 1c,d of the main text). Nonetheless, their concentration is too small to account for the difference between 47.8 and 50%. As discussed in Supplementary Note 1, these darker species could also correspond to K^+ ions sitting on (rare) aluminosilicate rings with three Al sites. Among the sample's other impurities, Fe^{2+} and Mg^{2+} do not substitute K^+, but Al^{3+} in the octahedral sheets[10]. Na^+ species replace K^+ and should appear fainter in nc-AFM because of their smaller ionic radius (see below for more comments on Na^+ ions). Other possibilities for the less-than-50% surface ion coverage are (iii) Missing OH^- groups from the subsurface; in this case, fewer K^+ ions would be needed for a charge-neutral system. (iv) Errors in the analysis of the nc-AFM images. In the images, ≈3−5% of the dark dots appear with fainter contrast than the rest (see the red arrow in Supplementary Fig. 7a for one example). It is not unreasonable that some of these faint species might be missed during the statistical evaluation. These species are likely to be Na^+ ions: They are expected to substitute K^+ and appear with fainter contrast due to their smaller ionic radius compared to K^+. Moreover, the nominal Na/(Na+K) ratio

according to the datasheet in Supplementary Fig. 5 is 8.7%, in the same order of the number of faint species counted in the nc-AFM images.

Average length of rows and row sections

Supplementary Figure 7c shows an analysis of the length of the straight ion rows (including straight sections of rows with kinks; excluding rows made of 2 ions; normalized to the number of rows in each image; error bars represent 95% confidence intervals calculated with a two-tailed Student's t-distribution from the standard error of the mean). Most of the straight rows or straight row sections are made of 3 K^+ ions (average excluding the rows made of 2 ions: 3.5±0.4 ions).

Autocorrelation analysis

An autocorrelation image (Supplementary Fig. 7f) was obtained from the mask of Supplementary Fig. 7e, showing the cations with normalized contrast. The autocorrelation image shows the probabilities of finding ions at given relative distances (see averaged probabilities in Supplementary Fig. 7g). In Supplementary Fig. 7f, regions of different occupation probabilities are marked by yellow circles to aid the eye. The six nearest neighbors ($d = 1$, ring closest to the center) have an occupation probability of 35.4±1.3%, lower than the average of 47.8±0.1%. Instead, the occupation probability of the ions at $d = 2$ and $d = \sqrt{3}$ (second ring) is slightly higher than average (49.9±1.4%). For ions farther away, it becomes close to average. The measured depletion at the nearest-neighbor positions is due to repulsion between the K^+ ions, which leads to alternating straight or kinked rows. These preferred geometries also lead to the probabilities of finding ions at d=2 and d=$\sqrt{3}$ greater than average. Assuming a perfect arrangement of infinite alternating rows (50% K^+ concentration), each ion within a row will have two out of the six nearest-neighbor sites occupied. The same is true for alternating zigzag (kinked) rows as shown in Fig. 3f of the main text. Thus, the probability of finding a K^+ nearest neighbor would be 1/3. The probability of finding a K^+ neighbor at $d = \sqrt{3}$ would be 1/3 and 2/3 for the ideal straight and zigzag configuration, and at $d = 2$ these probabilities would be 1 and 1/3, respectively. Since the experiments show both straight and kinked arrangements, the probabilities for K^+ neighbors at both $d = \sqrt{3}$ and 2 are between these two ideal cases. Since only short-range ordering is present, these probabilities are not far from the K^+ concentration. Notice that the occupation probabilities extracted from the experimental data fit reasonably well with those obtained from the MC-simulated distribution of Fig. 4e of the main text (see Supplementary Fig. 7g).

Comments on the Fourier Transform of the nc-AFM images

Notice that the background of the Fourier transform in Fig. 1e in the main text is not uniform, as it would be for a purely random distribution of K^+ ions. It displays a sort of star with maximum intensity within a rim at half the unit cell distance and arms stretching between the first-order periodicities. To unveil the origin of this background, the Fourier transform of the mask shown in Supplementary Fig. 7e was calculated (see Supplementary Fig. 8a; Supplementary Fig. 8b shows the corresponding power spectrum as a function of the spatial frequency, averaged over all azimuthal directions). This Fourier transform reproduces the features of the original nc-AFM image (apart from the decay towards high spatial frequencies, which is due to the finite lateral resolution of the nc-AFM image). Thus, the uneven background must originate from the positions of the ions and not from modulations in the AFM background or imaging artifacts.

The higher intensity within the rim between the zero- and first-order spots, also seen in the power spectrum of Supplementary Fig. 8b as a shallow and wide peak at spatial frequencies around 1 nm^{-1}, is because of the alternating rows formed at the surface (separation of 2 unit cells in real space). The peak is neither high nor sharp because of the lack of long-range order.

The depletion of the intensity close to the origin (and around the higher-order Fourier spots, which are essentially replicas of the origin) shows that the system avoids long-range coverage fluctuations, as expected for distributions dictated by electrostatic interactions. This interpretation is consistent with the fact that a similar Fourier transform is also obtained for the simulated K^+ lattice assuming electrostatic repulsion in the MC results in Fig. 4a (not shown). The ion distribution of Fig. 4e is different from that of Supplementary Fig. 7e, but it is still determined by electrostatic interactions. Hence it also minimizes long-range fluctuations in the local charge distribution.

In principle, one would expect to observe the variations of the diffuse background seen in the Fourier transform also in the LEED images. However, the additional diffuse phonon background around the diffraction spots[11] produces an increased intensity around the spots, which may hide the diffuse background caused by the K^+ distribution. This is probably the reason why early LEED works did not infer any hint of short-range order from their data[12].

Supplementary Note 6: Comment on LEED

Acquisition of LEED was attempted on the UHV-cleaved surface without success. The samples charge immediately when irradiated with the LEED electron beam. Patterns consistent with the

ones obtained by Müller et al.[12] could be obtained on samples that were not pristine, e.g., left in the UHV chamber for >24 h or exposed to small amounts of water at low temperature and warmed up to room temperature again. Apart from the trace elements already present after cleavage, the latter samples appeared clean within the detection limit of XPS[12].

Supplementary References

1. Herrero, C. P., Sanz, J. & Serratosat, J. M. Si, Al distribution in micas; analysis by high-resolution ^{29}Si NMR spectroscopy. *J. Phys. C Solid State Phys.* **18**, 13–22 (1985).

2. Properties and chemical composition of Mica Grade V1. https://www.tedpella.com/Vacuum_html/Mica_Grade_V1_Properties.aspx.

3. Loewenstein, W. The distribution of aluminum in the tetrahedra of silicates and aluminates. *Am. Mineral.* **39**, 92–96 (1954).

4. Xu, L. & Salmeron, M. An XPS and Scanning Polarization Force Microscopy Study of the Exchange and Mobility of Surface Ions on Mica. *Langmuir* **14**, 5841–5843 (1998).

5. Bhattacharyya, K. G. XPS Study of Mica Surfaces. *J. Electron Spectros. Relat. Phenomena* **63**, 289–306 (1993).

6. Sokolović, I., Schmid, M., Diebold, U. & Setvin, M. Incipient ferroelectricity: A route towards bulk-terminated $SrTiO_3$. *Phys. Rev. Mater.* **3**, 034407 (2019).

7. Sokolović, I. *et al.* Quest for a pristine unreconstructed $SrTiO_3$(001) surface: An atomically resolved study via noncontact atomic force microscopy. *Phys. Rev. B* **103**, 1–7 (2021).

8. Schneider, C. A., Rasband, W. S. & Eliceiri, K. W. NIH Image to ImageJ: 25 years of image analysis. *Nat. Methods 2012 97* **9**, 671–675 (2012).

9. Choi, J. I. J. *et al.* The growth of ultra-thin zirconia films onPd_3Zr(0001). *J. Phys. Condens. Matter* **26**, 225003 (2014).

10. Burzo, E. True micas. in *Landolt-Börnstein New Series III/27 I 5α* (ed. Wijn, H. P. J.) 108–291 (Springer; Berlin, Heidelberg, 2007). doi:10.1007/978-3-540-44748-1_6.

11. McKinney, J. T., Jones, E. R. & Webb, M. B. Surface Lattice Dynamics of Silver. II. Low-Energy Electron Thermal Diffuse Scattering. *Phys. Rev.* **160**, 523–530 (1967).

12. Müller, K. & Chang, C. C. Electric Dipoles on Clean Mica Surfaces. *Surf. Sci.* **14**, 39–51 (1969).
